# Comparison of Olfactory Genes in Two *Ectropis* Species: Emphasis on Candidates Involved in the Detection of Type-II Sex Pheromones

**DOI:** 10.3389/fphys.2018.01602

**Published:** 2018-11-14

**Authors:** Zhao-Qun Li, Xiao-Ming Cai, Zong-Xiu Luo, Lei Bian, Zhao-Jun Xin, Bo Chu, Yan Liu, Zong-Mao Chen

**Affiliations:** Key Laboratory of Tea Biology and Resource Utilization, Ministry of Agriculture, Tea Research Institute, Chinese Academy of Agricultural Sciences, Hangzhou, China

**Keywords:** transcriptomic analysis, olfaction gene, sex pheromone perception, *Ectropis grisescens*, *Ectropis obliqua*

## Abstract

The sibling species *Ectropis grisescens* and *E. obliqua* are the major chewing tea pests in China. A difference in sex pheromone components plays a central role in premating isolation in these two species. To investigate the mechanism of premating isolation in these two *Ectropis* species, we sequenced the transcriptomes of the antennae of female and male *E. obliqua* individuals and performed phylogenetic analyses, abundance analyses, and tissue expression profile analyses to compare the olfactory genes involved in the detection of sex pheromones. A total of 36 odorant-binding proteins (OBPs) and 52 olfactory receptors (ORs) were identified in *E. obliqua*. Phylogenetic analyses showed that EoblOBP2, 3, and 25 were grouped in the pheromone-binding protein clade with EgriOBP2, 3, 25, and another lepidopteran PBP. EoblOR25 and 28 were grouped with EgriOR25, 28, and pheromone receptors for the detection of Type-I sex pheromone components. EoblOR24, 31, 37, and 44 were grouped with EgriOR24, 31, 37, and 44. All of these 4 EoblORs and 4 *EgriORs* showed higher abundance in male antennae than in female ones. Therefore, OBP2, 3, 25 and OR24, 31, 37, 44 of *E. grisescens* and *E. obliqua* might be responsible for sex pheromone component detection. However, the sequences of these genes in *E. grisescens* and *E. obliqua* were more than 90% identical. This indicates that these orthologous genes might play similar roles in the detection of sex pheromones. In contrast, the observed *OBPs* and *ORs* differed in abundance between the antennae of the two *Ectropis* species. Therefore, we speculate that these two *Ectropis* species use the different transcript levels of PRs to differentiate sex pheromone components. The results of the present study might contribute in deciphering the mechanism for premating isolation in these species and may be of use in devising strategies for their management.

## Introduction

The tea geometrid, *Ectropis obliqua*, is a notorious chewing pest in the tea plantations of China (Ma et al., 2016b), and use of the *E. obliqua* nucleopolyhedrosis virus (EoNPV) preparation is an effective management strategy for its control. The susceptibility of tea geometrids collected from Quzhou, Zhejiang province to EoNPV is reported to be about 724.5-fold higher than in those collected from Yixing, Jiangsu province (Xi et al., 2011). Based on the assessment of external morphology, molecular biology, and interspecific hybridization, these two geographical populations were shown to be two different *Ectropis* species, namely *E. grisescens* and *E. obliqua* (Lepidoptera: Geometridae) (Jiang N. et al., 2014; Zhang G.H. et al., 2016). Because of morphological similarities, these two *Ectropis* species were mischaracterized as a single tea geometrid species, *E. obliqua*.

In moths, courtship and mating behaviors are regulated by sex pheromones, which play crucial roles in reproduction and are associated with reproductive isolation (Wyatt, 2009). The sex pheromones in both *E. grisescens* and *E. obliqua* were reported to be (Z,Z,Z)-3,6,9-octadecatriene (Z3,Z6,Z9-18:H) and (Z,Z)-3,9-cis-6,7-epoxy-octadecadiene (Z3,epo6,Z9-18:H) and were present at similar ratios (Ma et al., 2016c; Yang et al., 2016), which is unusual for sibling species occurring in the same region. The female sex pheromones of the two *Ectropis* species were reexamined in order to clarify how these two geometrids maintain premating isolation (Luo et al., 2017). The results showed that Z3,Z6,Z9-18:H and Z3,epo6,Z9-18:H were the sex pheromones of *E. grisescens*, whereas Z3,Z6,Z9-18:H, Z3,epo6,Z9-18:H, and (Z,Z)-3,9-cis-6,7-epoxy-nonadecadiene (Z3,epo6,Z9-19:H) were the sex pheromones of *E. obliqua*. Thus, the presence or absence of Z3,epo6,Z9-19:H may be the major determinant for premating isolation of these two *Ectropis* species. Moth sex pheromone components are classified into three groups: Type-I, Type-II, and miscellaneous type (Ando et al., 2004). Type-I sex pheromone components are composed of unsaturated compounds with C_10_–C_18_ straight chain unsaturated alcohols, aldehydes, or acetate esters. Type-II sex pheromone comprise unsaturated hydrocarbons and epoxy derivatives with a C_17_–C_23_ straight chain (Millar, 2000; Ando et al., 2004). Both *E. grisescens* and *E. obliqua* thus produce Type-II sex pheromone components.

Previous studies have shown that both soluble binding proteins and membrane-bound receptors are used in the detection of sex pheromones in moth (Leal, 2012).The odorant-binding proteins (OBPs) and water-soluble carriers are thought to aid in the capture and transport of odorants and pheromones to their receptors (Pelosi et al., 2014), and the pheromone-binding proteins (PBPs), a sub-class of OBPs, are thought to enhance the solubility of lipophilic Type-I sex pheromone components and deliver them to the membrane-bound receptors (Zhou, 2010; Sun et al., 2013; Jin et al., 2014). Sex pheromone receptors (PRs), a subfamily of odorant receptors (ORs), are specifically activated by Type-I sex pheromone components and have been widely studied in lepidopteran insects (Jiang X.J. et al., 2014; Zhang et al., 2014; Chang et al., 2015). In addition to PBPs and PRs, GOBP2 is also thought to be involved in the detection of Type-I sex pheromones (Liu et al., 2015). The analysis of the molecular mechanisms for the olfactory detection of sex pheromones in *E. grisescens* and *E. obliqua* might contribute to decipher the strategy for premating isolation in these two *Ectropis* species.

Less is known about the perception mechanism of Type-II pheromone components (Zhang D.D. et al., 2016). In our previous studies, we sequenced the antennae transcriptomes of *E. grisescens*, and identified 40 OBPs and 59 ORs, including an OR attuned to *E. grisescens* sex pheromone (Li et al., 2017). Although 24 OBPs and 4 ORs were identified from the leg transcriptome of *E. obliqua* (Ma et al., 2016a), the gene numbers were far different from *E. grisescens*. We therefore sequenced the transcriptomes of the antennae, the principal olfactory organs, of female and male *E. obliqua* individuals, and performed analyses of phylogeny, abundance, and tissue expression profile to compare the olfactory genes involved in sex pheromone detection in the two species.

## Materials and Methods

### Insect Rearing and Tissue Collection

Individuals of *E. grisescens* and *E. obliqua* were originally collected from the Experimental Tea Plantation of the Tea Research Institute, Chinese Academy of Agricultural Sciences (Hangzhou, Zhejiang, China). The larvae of the two species were accurately identified by comparing the *cytochrome c oxidase I* gene sequences and were reared on fresh tea shoots in different climate-controlled rooms under the same conditions (25 ± 1°C and 70 ± 5% relative humidity with a 14-h light:10-h dark photoperiod), enclosed in nylon mesh cages (70 cm × 70 cm × 70 cm). After pupation, the male and female pupae were separated based on their morphological characters and kept separately in cages for eclosion. After emergence, the adult moths were fed on a 10% honey solution. For transcriptome sequencing, antennae from 100 female and 100 male 2-day-old virgin *E. obliqua* individuals were collected in duplicates. For qRT-PCR analyses, a different sample of twenty 2-day-old virgin female and male *E. grisescens* and *E. obliqua* adults were used to collect antennae, heads without antennae, thoraxes, abdomen without the pheromone gland, legs, wings, proboscises, and pheromone glands. These tissues were immediately frozen and stored at −80°C until RNA isolation. Total RNA was extracted with TRIzol reagent (Invitrogen, Carlsbad, CA, United States). The quality of the RNA samples was assessed by agarose gel electrophoresis, NanoDrop (Thermo, Wilmington, DE, United States), and Agilent 2100 Bioanalyzer.

### cDNA Library Preparation, Illumina Sequencing, and *de novo* Assembly

The cDNA library construction and Illumina sequencing of the samples were performed at Novogene Bioinformatics Technology Co., Ltd. (Beijing, China). Poly adenylated mRNAs were isolated from 5 μg total RNA using oligo (dT) magnetic beads and were fragmented into short fragments in the presence of divalent cations in fragmentation buffer at 94°C for 5 min. Using these short fragments as templates, random hexamer primers were used to synthesize first-strand cDNAs. Second-strand cDNAs were generated using RNase H and DNA polymerase I. After end repair and adaptor ligation, the short sequences were amplified by PCR, purified with a QIAquick^®^ PCR Purification Kit (Qiagen, Venlo, Netherlands), and sequenced on the HiSeq^TM^ 2500 platform (San Diego, CA, United States). The *de novo* transcriptome assembly was carried out using the short-read assembly program, Trinity (r20140413p1) (Grabherr et al., 2011) based on the paired-end reads with default settings. The transcriptomic data were deposited in the NCBI/SRA database (SRR7757597 and SRR7757596). Transcripts longer than 150 bp were first aligned by BLASTX against protein databases (NR, Swiss-Prot, KEGG, and COG; *E*-value < 10^−5^) to retrieve the proteins with the highest sequence similarity to the unigenes along with their functional annotations. We then used Blast2GO (Conesa et al., 2005) for gene ontology (GO) annotation of the transcripts and WEGO software (Ye et al., 2006) to plot the results of the GO annotation.

### Expression of Transcripts and Differential Expression Analysis

Transcript abundance was calculated as reads per kilobase per million mapped reads (RPKM) method, which can eliminate the influence of different transcript lengths and sequencing discrepancies when calculating the abundance (Mortazavi et al., 2008). We used the following equation:

$$\mathrm{RPKM}(A)=\frac{C\times 10^{6}}{\frac{N\times L}{10^{3}}}$$

where *RPKM (A)* represents the RPKM value of the transcript *A*, *C* is the number of reads uniquely aligned to the transcript *A*, *N* is the total number of fragments uniquely aligned to all the transcripts, and *L* is the number of bases in the transcript *A*.

Genes showing differential expression between the two conditions/groups were detected using the DESeq R package (1.10.1) (Anders and Huber, 2010), which provides statistical routines to determine differential expression from digital gene expression data using a model based on negative binomial distribution. The resulting *P*-values were adjusted using Benjamini and Hochberg’s approach to control the false discovery rate. Genes with an adjusted *P*-value < 0.05 found using DESeq were considered to be differentially expressed.

### Identification of *E. obliqua* OBP and OR Genes and Sequence Analyses

Sequenced transcriptomes were annotated by combining the transcriptomes of the antennae from females and males, then searching against the non-redundant (NR) database using BLASTX with a cut-off *e*-value of 10^−5^. *EoblOBPs* and *EoblORs* were named according to sequence similarity with *EgriOBPs* and *EgriORs*.

### Sequence Alignment and Phylogenetic Analysis

The amino acid sequence alignments of EgriOBPs, EoblOBPs, EgriORs, and EoblORs were performed using ClustalX 2.0 (Larkin et al., 2007). To investigate the phylogenetic relationships of the OBPs and ORs between *E. grisescens*, *E. obliqua* and other insect species, we aligned them using MAFFT (E-INS-I parameter) (Katoh and Standley, 2013). The phylogenetic trees were constructed using PhyML 3.1 with LG substitution model to generate a maximum likelihood phylogenetic tree (Guindon et al., 2010). Finally, the trees were viewed and group edited with FigTree v1.4.2^1^.

### Quantitative Real-Time PCR Validation

The tissue expression patterns of *EgriOBPs*, *EgriORs*, *EoblOBPs*, and *EoblORs* in different tissues were measured using a qPCR method performed according to the minimum information for publication of quantitative real-time PCR experiments (Bustin et al., 2009). Total RNA was isolated using the SV Total Isolation System (Promega, Madison, WI, United States) according to manufacturer’s instructions. The quality and quantity of the RNA samples was assessed using agarose gel electrophoresis and NanoDrop (Thermo). Single-stranded cDNA templates were synthesized using the Reverse Transcription System (Promega) following manufacturer’s instructions. The qRT-PCRs were performed on a Bio-Rad CFX96 touch real-time PCR detection system (Bio-Rad, Hercules, CA, United States). The primers were designed using Beacon Designer 7.7 based on the *E. grisescens* and *E. obliqua* nucleotide sequences obtained from the transcriptome data (Supplementary Table S1). The reaction was performed as follows: 30 s at 95°C, followed by 40 cycles at 95°C for 5 s and 60°C for 34 s. Templates were diluted in a five-fold series of samples and were used to determine the amplification of primers. Each reaction was run in triplicate (technical repeats). Theguanine nucleotide-binding protein G(q) subunit alpha and glyceraldehyde-3-phosphate dehydrogenase genes of both *E. grisescens* and *E. obliqua* were selected as reference genes for the qPCR analysis. A blank control without template cDNA (replacing cDNA with H_2_O) served as the negative control. Each reaction included three independent biological replicates and was repeated three times (technical replicates). The relative transcript levels were calculated using the comparative 2^−ΔΔCq^ method (Livak and Schmittgen, 2001).

## Results

### Identification of *E. obliqua* OBP and OR Genes

**FIGURE 1:**
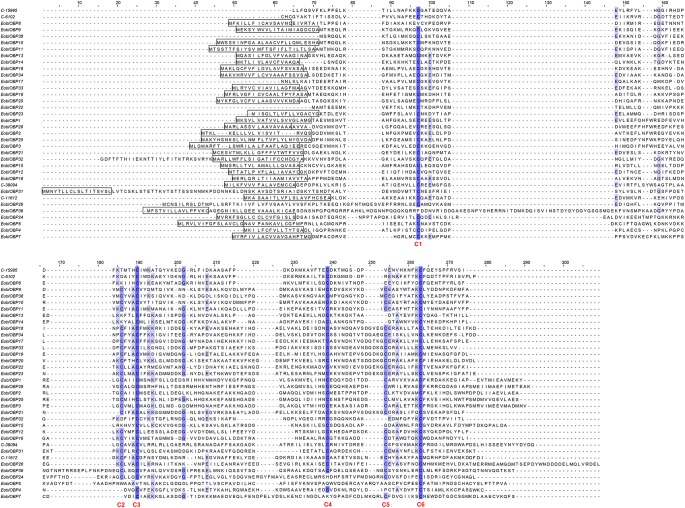
Alignment of amino acid sequences of EoblOBPs. Boxes indicate the predicted signal peptides; blue highlight indicates the conserved cysteine residues.

A total of 36 *EoblOBPs* were identified in the *E. obliqua* antennae transcriptome. Sequence analyses showed that 31 of the 36 *EoblOBPs* were full-length genes (Figure 1). Of the 36 EoblOBPs, EoblOBP8, 13, 14, 15, 32, C-15995, and C-6102 contained only four conserved cysteine residues. EgriOBP4, 5, and 7 contained more than six conserved cysteine residues. The other EoblOBPs contained six conserved cysteine residues. A total of 52 *EoblORs* were identified in the *E. obliqua* antennae, 37 of which contained a full-length open reading frame, and had a full-length of about 1200 bp. EgriOBP2, 3, 25 and EgriOR24, 28, 31, 27 44 were identified as candidate genes involved in detecting sex pheromones. The identities between the sequences of EoblOBP2 and EgriOBP2, EoblOBP3 and EgriOBP3, and EoblOBP25 and EgriOBP25 were 98.77, 98.84, and 97.65%, respectively, whereas those between the sequences of EoblOR24 and EgriOR24, EoblOR25 and EgriOR25, EoblOR28 and EgriOR28, EoblOR31 and EgriOR31, EoblOR37 and EgriOR37, and EoblOR44 and EgriOR44, were 96.04, 96.08, 90.68, 92.72, 99.29, and 90.68%.

### Phylogenetic Analyses of *E. obliqua* and *E. grisescens* OBP and OR Genes

**FIGURE 2:**
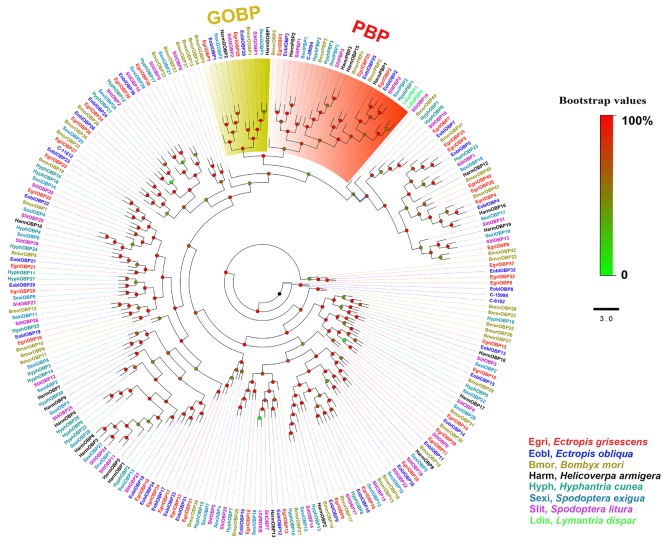
Phylogenetic analyses of EoblOBPs and EgriOBPs with the OBPs of other lepidopteran insects. GOBP and PBP subfamily are labeled. EgriOBP, OBPs from *Ectropis grisescens*; EoblOBP, OBPs from *Ectropis obliqua*; BmorOBPs, OBPs from *Bombyx mori*; HarmOBPs, OBPs from *Helicoverpa armigera*; HyphOBPs, OBPs from *Hyphantria cunea*; SexiOBPs, OBPs from *Spodoptera exigua*; SlitOBPs, OBPs from *Spodoptera litura*; LdisOBPs, OBPs from *Lymantria dispar*; Phylogenetic tree was constructed with PhyML3.1 using the maximum likelihood method.

**FIGURE 3:**
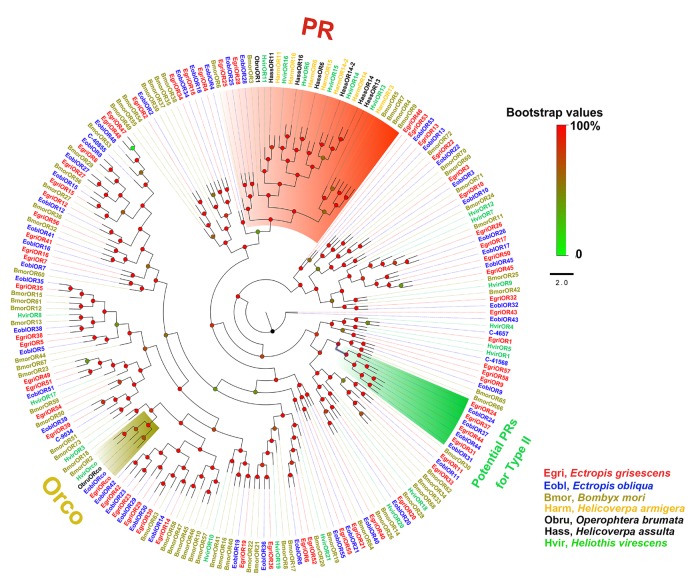
Phylogenetic analysis of EoblORs and EgriORs with ORs of other lepidopteran insects. The branches of Orco, PRs, and potential PRs for Type-II were labeled. EgriORs, ORs from *Ectropis grisescens*; EoblOR, ORs from *Ectropis obliqua*; BmorORs, ORs from *Bombyx mori*; HarmORs, ORs from *H. armigera*; ObruORs, ORs from *Operophtera brumata*; HassORs, ORs from *H. assulta*; HvirORs, ORs from *Heliothis virescens*. The phylogenetic tree was constructed with PhyML3.1 using maximum likelihood.

In the phylogenetic tree based on the OBP sequences, four EoblOBPs (EoblOBP2, 3, 25, and C-39094) were grouped in the PBP clade with EgriOBP2, 3, 25, and other lepidopteran PBP (Figure 2). The orthologs EoblOBP1 and 29 were in the GOBP group with their orthologs EgriOBP1 and 29 (Figure 2). The phylogenetic tree generated using the OR sequences showed that EoblORco was well clustered with EgriORco, ObruORco(ORco of *Operophtera brumata*), HvirORco(ORco of *Heliothis virescens*), and BmorOR2(ORco of *Bombyx mori*) with high bootstrap support (Figure 3). EoblOR25 and 28 were grouped with EgriOR25, 28, ObruOR1 (a PR for Type-II sex pheromone), and PRs for Type-I sex pheromone, including *B. mori*, *Helicoverpa armigera*, *H. assulta*, and *H. virescens* (Figure 3). EoblOR24, 31, 37, and 44 were grouped with EgriOR24, 31, 37, and 44 (Figure 3).

### Abundance of *E. obliqua* and *E. grisescens* OBP/OR mRNAs

**FIGURE 4:**
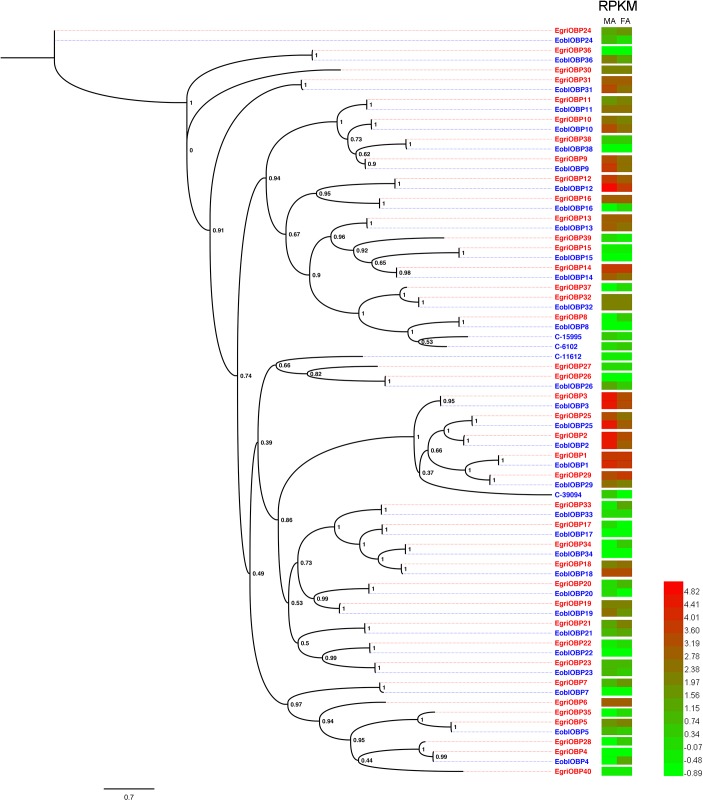
Phylogenetic analysis and abundance of OBPs in *Ectropis grisescens* and *E. obliqua*. MA, male antennae; FA, female antennae. The phylogenetic tree was constructed using PhyML3.1 with maximum likelihood.

**FIGURE 5:**
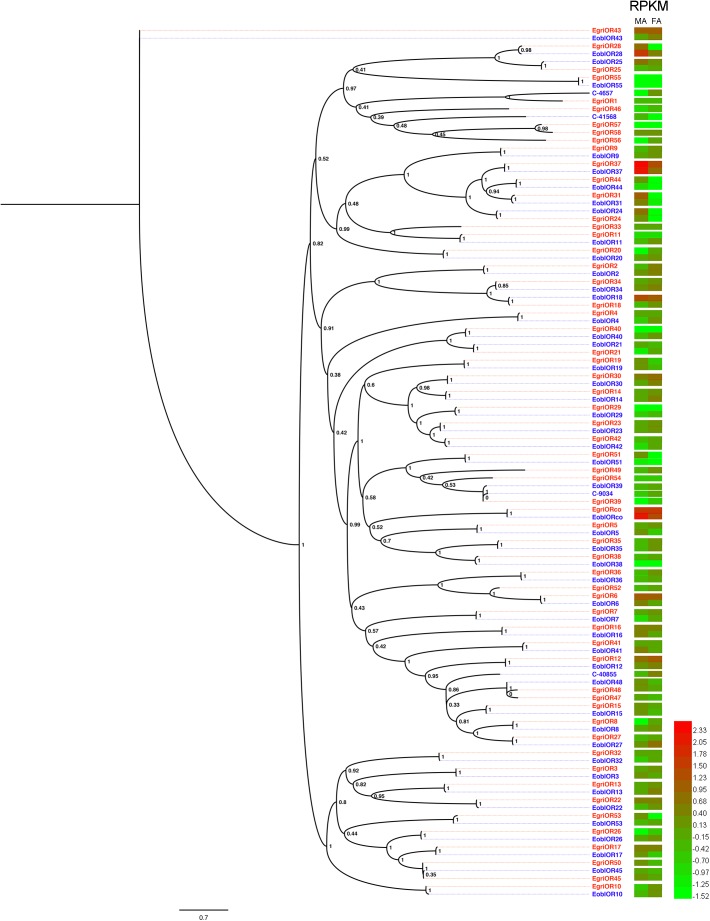
Phylogenetic analysis and abundances of ORs in *Ectropis grisescens* and *E. obliqua*. MA, male antennae; FA, female antennae. The phylogenetic tree was constructed using PhyML3.1 with maximum likelihood.

**FIGURE 6:**
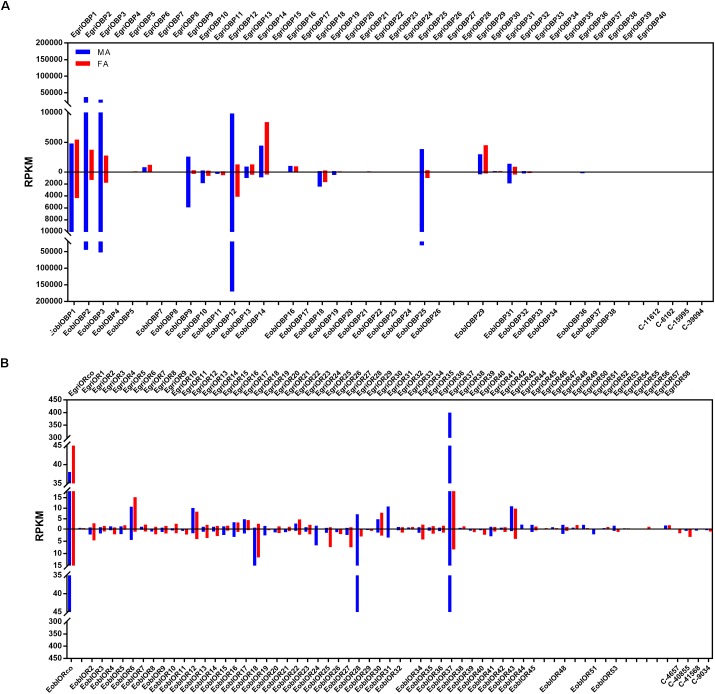
Expression levels of the selected *OBP* and *OR* genes in the antennae based on the RPKM values. **(A)** RPKM values of *OBPs* in the antennae of male and female *Ectropis grisescens* and *E. obliqua*. **(B)** RPKM values of *ORs* in the antennae of male and female *E. grisescens* and *E. obliqua*. FA, antennae of female individuals; MA, antennae of male individuals.

To compare the abundance of *E. obliqua* and *E. grisescens OBP/OR* in the antennae, we characterized their abundance by evaluating their RPKM values and combined these results with those of the phylogenetic analyses (Figures 4–6). We observed that most of the orthologs of *E. obliqua* and *E. grisescens OBP*/*OR* had similar expression levels in the antennae (Figures 4, 5). However, several orthologous genes that were more abundant in the antennae of male individuals showed different abundance in the antennae of the two *Ectropis* sibling species (Figure 6). *E. obliqua* and *E. grisescens OBP2*, *3*, *9*, *12*, and *25* showed higher transcription levels in the antennae of the male individuals than in those of the females (Figure 6). Among these 10 male antenna-biased *OBPs*, *EgriOBP2* and *3* were the two most abundant *OBPs* in the *E. grisescens* antennae, followed by *EgriOBP12*. However, *EoblOBP12* showed the highest transcript level in the antennae, which was more than four-fold higher than the *EoblOBP2* and *3* levels. Comparing the orthologous *OBP2*, *3*, *9*, *12*, and *25* of *E. obliqua* and *E. grisescens*, the RPKM values of *EoblOBP3*, *12* and *25* were respectively 1.8-, 17.3-, and 7.9-fold higher than their orthologs, *EgriOBP3*, *12* and *25*,. The transcript levels of *OBP2* and *9* were similar in *E. obliqua* and *E. grisescens*.

The analyses of the abundance of *EoblOR* and *EgriOR* mRNAs showed that *ORco* and *OR37* showed the highest expression levels in the antennae of *E. obliqua and E. grisescens* (Figure 6). The *E. obliqua and E. grisescens OR24*, *28*, *31*, *37*, and *44* were predominantly expressed in the antennae of the male individuals. However, the orthologs of these *ORs* showed different transcript levels in the two *Ectropis* sibling species. The RPKM values of *EoblOR24* and *28* were 4.2- and 8.0-fold higher than those of their orthologous genes, *EgriOR24* and *28*, respectively. On the other hand, *OR31*, *37*, and *44* showed higher expression levels in *E. grisescens* with RPKM values in the antennae of male *E. grisescens* being 3.2-, 2.6-, and 17.0-fold of the levels of their orthologs in the antennae of male *E. obliqua*.

### Tissue Expression Profile of *E. obliqua* and *E. grisescens* OBP/OR Genes

**FIGURE 7:**
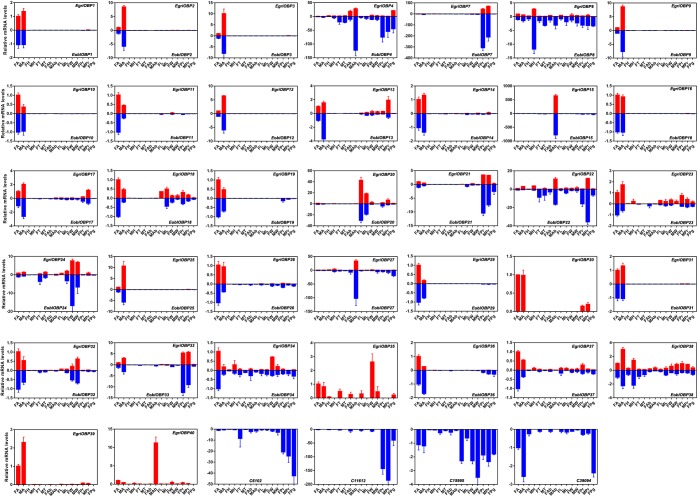
Tissue expression profiles of *EgriOBPs* and *EoblOBPs*. FA, antennae of female individuals; MA, antennae of male individuals; FH, head without antennae of female individuals; MH, head without antennae of male individuals; FT, thorax of female individuals; MT, thorax of male individuals; FAb, abdomen without pheromone gland of female individuals; MAb, abdomen of male individuals; FL, legs of female individuals; ML, legs of male individuals; FW, wings of female individuals; MW, wings of male individuals; FPr, proboscis of female individuals; MPr, proboscis of male individuals; Pg, pheromone gland.

Expression patterns of *E. obliqua* and *E. grisescens* OBP/OR genes in different adult tissues detected by qPCR showed that the orthologous genes of *EoblOBPs* and *EgriOBPs* had similar expression patterns (Figure 7). Most of the *EoblOBPs* and *EgriOBPs* were highly expressed in the antennae of both the female and male individuals. Among those, *E. obliqua* and *E. grisescens OBP2*, *3*, *9*, *12*, and *25* were expressed at higher levels in the antennae of males than females. *E. obliqua* and *E. grisescens OBP7*, *21*, and *33* were expressed in both female and male proboscises at relatively high levels. *EoblOBP20* and *EgriOBP20* were predominantly expressed in the legs of both sexes, and *EoblOBP24*, *EgriOBP24*, and *EgriOBP35* were mainly expressed in the wings of both sexes. *EoblOBP15* and *27*, and *EgriOBP15*, *27*, and *40* were highly expressed in male abdomens. The expression patterns of the other OBP genes were ubiquitous in most tested tissues, at relatively high levels.

**FIGURE 8:**
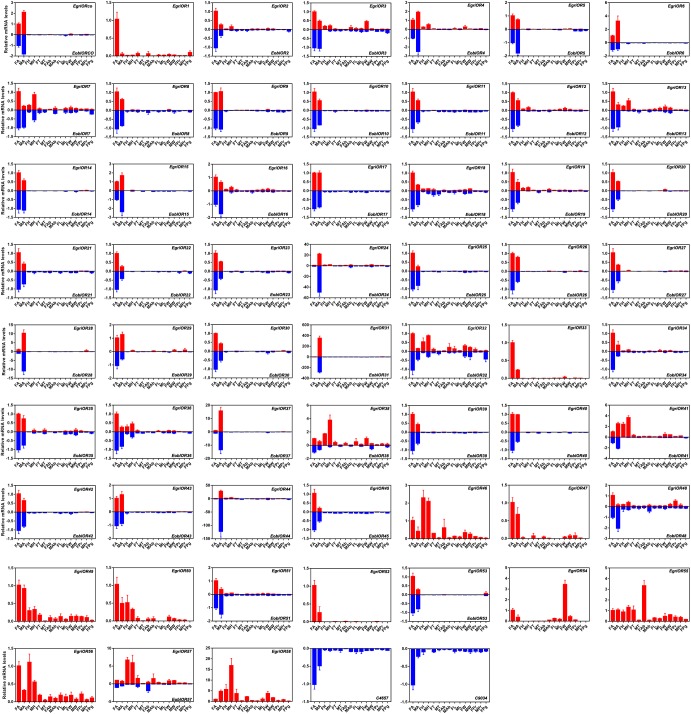
Tissue expression profiles of *EgriORs* and *EoblORs*. FA, antennae of female individuals; MA, antennae of male individuals; FH, head without antennae of female individuals; MH, head without antennae of male individuals; FT, thorax of female individuals; MT, thorax of male individuals; FAb, abdomen without pheromone glands of female individuals; MAb, abdomen of male individuals; FL, legs of female individuals; ML, legs of male individuals; FW, wings of female individuals; MW, wings of male individuals; FPr, proboscis of female individuals; MPr, proboscis of male individuals; Pg, pheromone gland.

The analyses of the expression profile of *ORs* showed that the orthologs of *EoblORs* and *EgriORs* also had similar profiles, as observed for *EoblOBPs* and *EgriOBPs* (Figure 8). Most of the *EoblORs* and *EgriORs* were mainly expressed in the antennae. Among the ORs showing an expression bias for the antenna, *E*. *obliqua* and *E*. *grisescens OR24*, *28*, *31*, *37*, and *44* were more highly expressed in the antennae of males than in those of females. *EoblOR7*, *32*, *41*, and *EgriOR7*, *32*, *41*, *57*, *58* were expressed in female and male heads at relatively high levels. *EgriOR54* was predominantly expressed in female wings, and *EgriOR55* was highly expressed in male abdomens.

## Discussion

Sex pheromone-induced behavior plays crucial roles in insect reproduction. The difference in the sex pheromone components of Z3,epo6,Z9-19:H may be the major determinant for premating isolation between these two *Ectropis* sibling species (Ma et al., 2016c; Luo et al., 2017). This difference in the sex pheromone components indicates that these two *Ectropis* sibling species might differ in the detection of sex pheromones, leading to premating isolation. We identified the candidate genes for detection of *E. grisescens* sex pheromones and analyzed the transcriptomes of the antennae of female and male individuals of *E. obliqua* to identify olfactory genes potentially involved in the perception of sex pheromones, for comparison with *E. grisescens*.

Insect PBPs are responsible for detecting the sex pheromone components in lepidopterans (Leal, 2012). In our study, we identified 36 *EoblOBPs* in *E. obliqua*. Generally, olfactory genes involved in detecting sex pheromones were expressed at higher levels in the male antennae than in female antennae. The abundance in antennae and tissue expression profiles showed that *E. obliqua* and *E. grisescens OBP2*, *3*, *9*, *12*, and *25* were predominantly expressed in the male antennae, with relatively high RPKM values. Among these, *EoblOBP2*, *3*, *25* and *EgriOBP2*, *3*, *25* were grouped in the PBP clade with another lepidopteran PBP. Therefore, *OBP2*, *3*, and *25* from *E. obliqua* and *E. grisescens* might encode the PBPs for Type-II pheromone components. However, the abundance of *OBP3* and *25* in antennae differed between *E. obliqua* and *E. grisescens*. The RPKM values of *OBP3* and *25* in *E. obliqua* were higher than in *E. grisescens*. On the other hand, the amino acid sequences of *OBP2*, *3*, and *25* in *E. obliqua* and *E. grisescens* were more than 97% identical, indicating that these three orthologous genes might have similar functions in binding and transporting sex pheromone components. Therefore, the difference in the transcript levels might be used to detect the difference in sex pheromone components of these two *Ectropis* sibling species. Unlike *OBP2, 3*, and *25*, *OBP12* was not grouped in the PBP clade, but it was the most abundant *EoblOBP* in the antennal transcriptome of male *E. obliqua* individuals. The RPKM value of *EoblOBP12* was 17.3-fold higher than that of *EgriOBP12*, indicating that *OBP12* might relate to sex pheromone perception in *E. obliqua* and *E. grisescens*. The GOBP2, another sub-class of OBPs, is reported to strongly bind sex pheromones in *S*. *litura* (Liu et al., 2015), *B. mori* (Zhou et al., 2009), and *S. exigua* (Liu et al., 2014). EoblOBP1 and EgriOBP1 were grouped in the GOBP2 sub-class. Consequently, we speculate that these two OBPs might be involved in the binding of sex pheromone components.

Insect PRs, a key sub-class of ORs, are specifically dedicated to the detection of sex pheromone components in the Lepidoptera (Jiang X.J. et al., 2014; Zhang et al., 2014; Chang et al., 2015). In our previous study, we found that EgriOR25 and 28 were grouped in the PR clade with PRs for Type-I sex pheromone components and four male antenna-biased EgriORs (EgriOR24, 31, 37, and 44) formed an independent group in the phylogenetic analysis (Li et al., 2017). Of these four male antenna-biased EgriORs, EgriOR31 responded robustly to Z3,epo6,Z9-18:H but weakly to Z3,Z6,Z9-18:H (Li et al., 2017). Because PRs are a conserved sub-class of OR, EgriOR24, 25, 28, 31, 37, and 44 might be potential PRs of *E. grisescens*. In present study, EoblOR25 and 28 were grouped with EgriOR25, 28, ObruOR1, and PRs of *B. mori*, *H. armigera*, *H. assulta*, and *H. virescens*, and EoblOR24, 31, 37, and 44 were grouped with EgriOR24, 31, 37, and 44. The results of tissue expression profiles indicate that *EoblOR24*, *25*, *28*, *31*, *37*, and *44* had similar expression patterns with *EgriOR24*, *25*, *28*, *31*, *37*, and *44*, with these more highly expressed in the male antennae than in other tissues. Therefore, the number of potential PRs that we identified in *E. obliqua* was the same as in *E. grisescens*. Moreover, the sequence identity matches of OR24, 25, 28, 31, 37, and 44 between *E. obliqua* and *E. grisescens* were greater than 90%. Among these, the identity between EoblOR37 and EgriOR37 was as high as 99.29%. The high degree of similarity in sequence identities and tissue expression patterns indicate that these orthologous genes might play similar roles in the detection of sex pheromones. The equal number and high identity of PRs between these two *Ectropis* sibling species implies that the differences in the detection of sex pheromones might occur at the transcription state.

We determined the RPKM values of *OR24*, *28*, *31*, *37*, and *44* to characterize their abundance in the antennae of *E. obliqua* and *E. grisescens*. These values showed that the abundance of these *ORs* in the antennae differed between *E. obliqua* and *E. grisescens*. *EoblOR24* and *28* showed much higher transcript levels than *EgriOR24* and *28*, while, the transcript levels of *EoblOR31*, *37*, and *44* were much lower than those of *EgriOR31*, *37*, and *44*. The differences in the transcript levels of *OR24*, *28*, *31*, *37*, and *44* between *E. obliqua* and *E. grisescens* might relate to the recognition of different sex pheromones. Because *E. obliqua* and *E*. *grisescens* (Jiang N. et al., 2014; Zhang G.H. et al., 2016) underwent sympatric speciation, the males of these two *Ectropis* sibling species must recognize females of their own species by correctly differentiating the sex pheromones of *E. obliqua* and *E*. *grisescens*. This means that the *E*. *grisescens* male can detect Z3,epo6,Z9-19:H, the sex pheromone of *E. obliqua*. Therefore, it is understandable that these two *Ectropis* sibling species have an equal number of PRs with high identities, and regulation of the transcript levels of PRs might be selected by the two species to differentiate the difference in their sex pheromone components. Further research about the functions of *E. obliqua* and *E. grisescens* OBP1, 2, 3, 9, 12, and 25 and OR24, 25, 28, 31, 37, and 44 in the detection of sex pheromones is required to understand how these two *Ectropis* sibling species recognize the differences of their sex pheromone components.

## Author Contributions

Z-QL and Z-MC conceived and designed the experiments. Z-QL performed the experiments and wrote the manuscript. Z-QL, X-MC, Z-XL, LB, Z-JX, BC, and YL analyzed the data. All authors reviewed the final manuscript.

## Conflict of Interest Statement

The authors declare that the research was conducted in the absence of any commercial or financial relationships that could be construed as a potential conflict of interest.

## Footnotes

1 http://tree.bio.ed.ac.uk/software/figtree/
